# Cure of Hæmorrhoids by the Hypodermic Syringe

**Published:** 1879-05

**Authors:** Edmund Andrews

**Affiliations:** Prof. of Surgery in the Chicago Medical College; No. 6, Sixteenth St., Chicago, Ill.


### ARTICLE V.

### THE CURE OF HÆMORRHOIDS BY THE HYPODERMIC SYRINGE.

By EDMUND ANDREWS, A.M., M.D., Prof. of Surgery in the Chicago Medical College.

In a former number of this journal, I published the secret method of certain itinerant "Pile Doctors," and asked for information from all physicians who had any knowledge of the practical results of the treatment. This request, supplemented by other inquiries, has brought me responses from about 300

physicians, and given me more or less knowledge of the results of over 3,300 cases treated by the new method. From the material thus collected I am able to present the following history:

In the year 1871, there lived in the village of Clinton, Ill., a physician named Mitchell. His practice being small, he employed his superabundant leisure in planning a new treatment for hæmorrhoids. He was a good thinker, and soon conceived the idea of charging a hypodermic syringe with equal parts of carbolic acid and olive oil, and injecting the contents into the hæmorrhoidal tumors. He also devised another and totally different plan, which was to take two large needles with triangular points, like those used by saddlers, and then to pick the piles to pieces little by little with the needles. Mitchell himself is said to prefer the needle operation, and several others have adopted it from him, but the plan of injections has proved by far the most popular with others, and has recruited, in a quiet way, a surprising number of operators. The secret was sold from man to man, and the price and enthusiasm rose simultaneously. "State and county rights" to practice it, were vended at high rates, reaching in one instance the sum of $3,000. Regular physicians abandoned their practice, and even mortgaged their property for money with which to buy the secret, and set themselves up as itinerants, while ignorant laymen joined in the rush until they filled the whole West with their clamor, and at last whitened the sands of the Pacific shores with their hand-bills.

The chief managers of the business settle in the larger towns in the winter, where they advertise and practice, but as Spring advances to the time when the wild geese begin to fly, they feel the migratory instinct, and go from place to place, selling the secret to all who will buy it, and operating meanwhile on the people of the farms and villages. In this way they have treated more than 10,000 patients in the States west of the Alleghany mountains. A secret so extensively sold, always gets out. Three years ago I discovered and published it, thus putting a check on the business of selling, and induced large numbers of the regular profession to try the plan among their patients. These physicians have furnished me my best information, but I also opened communication with the principal itinerants themselves, and induced several of them to come out frankly and tell what they knew, and by checking one statement against another, was able to sift out pretty well the few attempts at deception.

Mitchell's original plans have excited widely extended thought and experimentation among his followers, so that his two methods have branched out into numerous varieties. The original injection seems to have consisted of equal parts of crystallized carbolic acid and olive oil. The operator exposes the piles to view, and smears the anus with an ointment to prevent smarting in case the fluid should chance to drop; he then takes a sharp-pointed hypodermic syringe, charged with the carbolized liquid, and slowly throws a few drops into one of the piles. The pipe is left in the puncture a few moments to prevent the fluid from running out, and to allow it to become fixed in the tissue. The pile turns white, and in the most successful cases withers away without pain, suppuration or sloughing. Only one pile is treated at a time, and about a week is allowed between the sessions, until all are cured. The itinerants often advertise their method as "painless," but as a matter of fact only about one patient in four gets anything like exemption from pain. Most of them suffer a sharp temporary smarting, and a few have a terrible and prolonged agony. The majority are cured, however, without interrupting their business.

The original plan has sprouted into numerous varieties. Instead of using olive oil as the excipient, many use glycerine. Then every operator has his favorite degree of strength. Several claim that the stronger the fluid the better it is, and actually inject crystals of carbolic acid melted by heat, while others use mixtures varying in strength all the way down the scale, until we find Dr. Weir, of New York, experimenting with one part of acid to 20 or 30 parts of the solvent. The dose injected varies in like manner. Some advocate great caution, and only put in from one to three drops, while others cram the pile with a syringe full, and seek to make it suppurate or slough. I find two men using creosote instead of carbolic acid, and several add anodynes, such as morphine, chloral, or iodoform. Ergotine is also a favorite injection, and a great number of mixed formulæ have been imparted to me, some of them containing five of six ingredients. Mr. Colles, of Dublin, injects muriated tincture of iron. Dr. Hill, of Bloomington, Ill., and Dr. Drake, of Hastings, Mich., use the iron persulphate, while others have tried tannin, chromic acid, tincture of iodine, etc. One itinerant, who writes in a straightforward, manly tone, says that he has experimented on almost every coagulating agent in the vegetable and mineral kingdoms. His preference is for the strongest carbolic acid. He adds the following remarks: "The difficulty with all remedies except carbolic acid is the suppuration being limited to a small portion of the tumor, or, like the preparations of iron, causing it to swell and become very painful. Carbolic acid is, so to speak, used up in cooking the blood throughout the entire tumor. The appearance of the pile in from five to twenty seconds shows such to be the fact. Suppuration takes place in three or four days, with sloughing. No danger of hæmorrhage."

The results of these various methods of treatment, may be summed up as follows: In the first place the needle operation has never become a favorite. I can learn of only five persons who make much use of it. The following case was probably treated in that way. The patient, a plethoric man of 45 years, went to a quack in Chicago, and as the result, a varicose hæmorrhoidal vein was widely opened. He says the blood gushed out freely, but after some trouble was arrested through the application of means not clearly understood by him. He then returned home in great agony, and sent for his family physician, who in turn called me in council. The family physician took off sundry cloths and compresses, and found a large opening in a vein plugged with a vial cork. The quack, I presume, tore open the thin walls of a dilated vein, and being driven to his wits' end by the gush of blood, finally concluded to cork up his patient like a demijohn.

My informants agree that the injection method seldom fails to cure the disease, but they report some serious disasters. The writers know of about 3,304 cases treated in their vicinities by these methods, and though they cannot always give exact numbers and details, yet the circumstances are such that a case of rapid death from the treatment, could not be concealed, though minor troubles, such as pain, sloughing, etc., might frequently escape their notice. It is probably, therefore, that the list of deaths is pretty complete, while the figures giving the minor accidents, are too small.

LIST OF ACCIDENTS.

| | |
|---|---|
| Deaths | 9 |
| Embolism of the liver (suspected) | 8 |
| Very dangerous hæmorrhage | 5 |
| Less " " | 5 |
| Carbolic acid poisoning (recovered | 1 |
| Sloughing (generally but not always confined to the piles) | 23 |
| Abcess (of the liver) | 1 |
| Severe inflammation | 10 |
| Violent pain | 83 |
| Stricture of the rectum | 2 |
| Permanent impotence | 1 |
| Long sickness (2 weeks to 6 months) | 6 |
| Relapsed | 7 |
| Failed of cure of piles | 11 |
| Sundry other accidents | 12 |
| | 184 |

Cases of sloughing and suppuration of the piles, are innumerable. Some itinerants use strong injections with the express purpose of producing these results, deeming that the plan of causing them to atrophy without suppuration, lacks certainty and permanence.

The list shows that while the deaths are so few that the risk is no greater than in other modes of treatment, yet the minor accidents are very numerous. The imperfection of the reports renders a thorough s udy of the accidents impossible, but the following informmation has been gleaned. One of the deaths was caused by inflammation followed by immense abscesses, erysipelas and pyæmia. The patient died on the fifth day.

Another death apparently resulted from embolism of the liver. That viscus nearly ceased its function; the stools were light-colored and scanty, the skin yellow, and all the lymphatic glands of the groin, axillæ, and neck became enlarged. A full dose of calomel always brought temporary improvement, but no permanent benefit. The patient lingered along, and died unrelieved, about one hundred days after the operation. The next fatal case was that of a man 84 years of age. The person who injected the pile, said it was "very large and very deeply seated." It was suspected that he mistook the enlarged prostate for a hæmorrhoid. Be that as it may, the patient was attacked with violent pain and retention of urine, and though relieved by the catheter, died the third day. There is no proof however, that the prostate was injected, nor that it would be fatal if it were.

The fourth death was also attributed to injecting the prostate, but no symptoms are given.

The five remaining deaths are so vaguely reported, that I am unable to give any particulars about them. It is possible that three of the reports refer to the same patient, and ought to be counted as one, in which case the whole number of deaths is only seven. This number of fatal results in 3,300 cases, treated often in the most reckless and ignorant manner, is certainly not large, and tends to show that the injection method is as safe as any other, so far as life is concerned.

The same relative immunity appears respecting hæmorrhage. Five dangerous cases of it are reported, but in most, if not all of them, it occurred from the foolhardy practice of allowing the patient to take long rides and walks when he should have been in bed; but even with all this imprudence the hæmorrhagic cases are fewer than occur after the use of the clamp and the ligature. Allingham reports more instances of hæmorrhage after his favorite operation, the ligature, than I can find among all these cases of injection.

The chief objection of the profession to this operation, has been the fear of embolism. The two lower pairs of hæmorrhoidal veins send their blood by the route of the internal iliacs to the heart, but they are small, while the upper pair is much larger, and carries the great mass of the blood of the hæmorrhoidal plexus to the liver, hence we should expect that embolism, if it occurred at all, would be of the latter organ. The facts agree with the indications of the anatomy, for not a single case is reported of a sudden death, such as would proceed from clots swept to the heart and lungs, but there are eight instances of suspected embolism of the liver; only one of them died, and there was no post-mortem examination, so that possible proof is wanting.

The first is the fatal case of liver trouble already described. The second was marked by an abscess of the liver, but the patient recovered. In the third case the patient was attacked one hour after the injection with severe pain in the liver. After some time the pain was relieved, and no further trouble followed, but the physician feared to repeat the injection. In the remaining cases it is simply reported to me that the patients after operation, were attacked with disease of the liver, but did not die; no particulars were given. It is probable that in a portion of the cases the liver disease pre-existed, and was the cause of the piles and not the consequence of the operation. On the whole, there does not appear to be any decided danger of embolism, if the case is carefully handled. I may mention here, that Dr. Whitmire of Metamore, Ill., practices tamponing the upper part of the rectum for 24 hours, to prevent any emboli from moving in that direction.

Sloughing and suppuration of the piles generally follows large and concentrated injections, but not the small and dilute ones. A few cases only of extensive abscesses have occurred.

The most frequent of all accidents is the occurrence of severe pain. The verge of the anus is extremely sensitive, and injections put in near that circle are liable to produce fearful distress, but above the verge, the sensibility rapidly diminishes, so that much less suffering is entailed by the injection of internal piles. In about one-fourth of the patients the pain is very slight. Dr. Weir of New York injected two series of patients, one with strong, and the other with weak carbolized solutions, using in the latter only one part of carbolic acid to ten, twenty or thirty of the excipient. He found that the pain and the abscesses followed the use of the strong injections, but were escaped when weaker ones were imployed.

The remaining accidents in the list are not peculiar to this operation, nor greater in number than occur in other methods. The operation was a new one, and its conditions of safety were unknown. When we consider that many of the operators were ignorant blockheads, with no qualifications for the business except a bottle of carbolic acid and a hypodermic syringe, and with no idea of efficiency but to distend the hæmorrhoidal plexus, with all the liquid caustic they could get into it, we shall not be surprised at discovering a few deaths and a number of minor accidents. Had the method itself not been an unusually safe one, they would certainly have slaughtered their scores of victims, for the difference is world-wide between their ignorant injecting, and cautious, scientific surgery. If the following rules be observed I believe that the method of treatment by hypodermic injection will be less painful than any other, and equally safe.

1. Inject only internal piles.

2. Use diluted forms of the remedy at first and stronger ones only when these fail.

3. Treat one pile at a time, and allow from four to ten days between the operations.

4. Inject from one to six drops, having smeared the membranes with cosmoline to guard against dripping. Inject very slowly and keep the pipe in place a few moments to allow the fluid to become fixed in the tissues.

5. Confine the patient to bed the first day, and also subsequently if any severe symptoms appear. Prohibit any but very moderate exercise during the treatment.

Under all treatments as well as when left without treatment, piles are subject to possible hæmorrhage. Allingham gives the following method of applying the tampon where the bleeding vessel cannot be found promptly, aud controlled by other means. He takes a good-sized sponge and fastens a strong double string through its center. (He prefers a bell-shaped sponge inserted with the open end downward.) Having pushed the sponge up the rectum some inches beyond the bleeding point, he fills the parts below with cotton dusted with powdered alum or persulphate of iron, and ties a stick across the finished tampon with the double string. By turning the stick like the handle of a gimlet, he twists and tightens the string, forcing the tampon firmly up against the sponge and causing it to spread laterally and compress the bleeding vessels. He advises the insertion of a large catheter with the tampon to give exit to the flatus. By the help of opiates the tampon is often tolerated several days.

My final conclusion is that the wild itinerants of the prairies have really made a valuable contribution to scientific knowledge, and that the cautious injection of hæmorrhoids with carbolized solutions will remain as one of the permanent operations of surgery.

The number of physicians who have contributed to this investigation is so large, that it is impossible to give separate credit to each one, but I return them collectively my thanks, and especially to Dr. J. Bussy, of Raub, Ind., who was kind enough to write to over thirty medical men in my behalf.

The literature of this subject is very scanty, and is mostly covered by the following references:

THE CHICAGO MEDICAL JOURNAL AND EXAMINER, October, 1876, article by Prof. E. Andrews.

The *N. Y. Medical Record*, Vol. XI., p. 112.

The *Maryland Medical Journal*, June, 1878.

The *London Lancet*, pp. 228, 1877, Article by H. A. Reeves.

The *Philadelphia Medical and Surgical Reporter*, July 6, 1878.

The *Cincinnati Eclectic Medical Journal.* Three articles in 1874, 1877, and in December, 1878.

The *Toledo Medical and Surgical Journal*, November, 1877. Article by Prof. J. A. Pooley.

The *Louisville Medical Journal*, August, 1878. Article by Dr. J. M. Matthews.

The *N. Y. Medical Journal*, March, 1879. Report by R. F. Weir, M.D., to the Therapeutical Society.

The *American Medical Bi-Weekly*, April 27, 1878. Paper of Dr. J. M. Matthews, before the Kentucky State Medical Society. Also in the same journal, Feb. 16, 1878, a paper read before the same society by A. B. Cook, A.M., M.D.

The St. Louis *Clinical Record*, April, 1878. Article by S. B. Hauts, M.D.

Ashurst's Surgery, 2d edition, p. 831.

"Hæmorrhoids or Piles, etc.," a pamphlet by E. J. Fraser, M.D., San Francisco, Cal. No date.

*Journal de Médecine et de Chirurgie*, Paris. Tome I. Mars, 1879.

NO. 6, SIXTEENTH ST., CHICAGO, ILL.

